# Exploring Antioxidant and Enzymes (A-Amylase and B-Glucosidase) Inhibitory Activity of *Morinda lucida* and *Momordica charantia* Leaves from Benin

**DOI:** 10.3390/foods9040434

**Published:** 2020-04-04

**Authors:** Michaelle Chokki, Mihaela Cudălbeanu, Cheikna Zongo, Durand Dah-Nouvlessounon, Ioana Otilia Ghinea, Bianca Furdui, Robert Raclea, Aly Savadogo, Lamine Baba-Moussa, Sorin Marius Avamescu, Rodica Mihaela Dinica, Farid Baba-Moussa

**Affiliations:** 1 Laboratoire de Microbiologie et de Technologie Alimentaire, FAST, Université d’Abomey-Calavi, ISBA-Champ de foire, Cotonou 01BP: 526, Benin; 2 Laboratoire de Biochimie et immunologie Appliquées (LABIA), Ecole Doctorale Sciences et Technologies, Université Joseph KI-ZERBO, 03 BP 7021 Ouagadougou 03, Burkina-Faso; zcheik@yahoo.fr (C.Z.); alysavadogo@gmail.com (A.S.); 3 Department of Chemistry, Physics and Environment, “Dunarea de Jos” University of Galati, 47 Domneasca Street, 800008 Galati, Romania; mihaela.cudalbeanu@ugal.ro (M.C.); dahdurand@gmail.com (D.D.-N.); ioana.ghienea@ugal.ro (I.O.G.); 4 Laboratory of Biology and Molecular Typing in Microbiology, Department of Biochemistry and Cell Biology, University of Abomey-Calavi, Faculty of Sciences and Techniques, Cotonou 05BP1604, Benin; laminesaid@yahoo.fr; 5 Department of Chemistry, Faculty of Natural Sciences, Imperial College London, London SW7 2AZ, UK; robert.raclea@yahoo.com; 6 Department of Organic Chemistry, Biochemistry and Catalysis, Faculty of Chemistry, University of Bucharest, 90-92 Soseaua Panduri, Bucharest, Romania; sorin_avramescu@yahoo.com; 7 University of Agronomic Science and Veterinary Medicine, 59 Marasti Blvd, 011464 Bucharest, Romania

**Keywords:** antioxidant, diabetes mellitus, plant extracts, HPLC, DPPH, β-Carotene–linoleate bleaching, Benin

## Abstract

Background: *Momordica charantia* Linn. (*Cucurbitaceae*), the wild variety of bitter melon and *Morinda lucida* Benth (*Rubiaceae*) were commonly used as a popular folk medicine in Benin. This research focused to measure the antioxidant and enzyme inhibitory effects of *M. charantia* and *M. lucida* leaves and their antidiabetic activity. Methods: Antioxidant activities were evaluated by micro-dilution technique using DPPH free radical scavenging activity and β-carotene-linoleate bleaching assay. The α-amylase inhibition assay was carried out utilizing the 3,5-dinitrosalicylic acid procedure, while β-glucosidase inhibition assay was demonstrated using as substrate *p*-nitrophenyl-β-D-glucopyranoside (PNPG). HPLC-DAD analysis was realized using a high-performance liquid chromatography systems with diode-array detector, L-3000. Results: Chlorogenic acid, epicatechin, daidzein, rutin, naringin, quercetin, naringenin and genistein were identified as polyphenol compounds in the both plants extract. Dichloromethane and ethyl acetate extracts showed a good α-amylase inhibitory activity (56.46 ± 1.96% and 58.76 ± 2.74% respectively). *M. lucida* methanolic extract has shown IC_50_ of 0.51 ± 0.01 mg/mL, which is the lowest for DPPH scavenging activity. *M. lucida* dichloromethane extract showed the highest inhibitory capacity of β-glucosidase activity (82.11. ± 2.15%). Conclusion: These results justify some traditional medicinal uses of both plants. The purified fractions could be used in future formulations, possibly incorporated in functional foods to combat certain diseases.

## 1. Introduction

Medicinal plants are used throughout the world in the preventive or curative treatment of several diseases [1]. Despite the progress of modern medicine, which for the most part uses synthetic products and the latest generation techniques, there is an alarming evolution of several chronic diseases including microbial, parasitic, viral infections, cancers, oxidative stress and many more. *Diabetes mellitus* (DM) is a chronic metabolic illness featured by hyperglycemia. It is presently one of the costliest and difficult chronic diseases and is a condition that is rising in epidemic proportions overall the world [2]. The management of diabetes in the absence of any side effects is up to this time a challenge to the medical system [3]. The therapeutic approaches for reducing postprandial hyperglycemia is to hinder the glucose absorption by the inhibition of carbohydrate-hydrolyzing enzymes, such as α-amylase [4]. Thus, the slowness of the α-amylase action by inhibitors could be one of the most efficient approaches to control Type 2 DM.

Apart from amylases, glucosidases are enzymes that catalyze the cleavage of glycosidic bonds in oligosaccharides or glycoconjugates. β-glucosidase is an enzyme of the hydrolases group, related to be implicated in the glycoproteins processing. Therefore, the quest for new β-glucosidase inhibitors is significant due to their therapeutic potential in the treatment of diabetes, human immunodeficiency virus infection, metastatic cancer, lysosomal storage disease, etc. [5]. Many studies have been performed on anti α-glucosidase action of natural products while investigation on their anti β-glucosidases properties are completely ignored in spite of their important role in diabetes and other diseases [6].

It is known that diabetes and its complications are connected with free radical mediated cellular injury [7]. In recent years, the antioxidants have been utilized for the prevention of cardiovascular disease, cancer and diabetes. The free radicals’ role in the human disease pathogenesis including cancer, aging and atherosclerosis has been recognized [8]. Electron acceptors, such as molecular oxygen, respond fast with free radicals to become radicals themselves, also referred to as reactive oxygen species (ROS). ROS are associated with cellular and metabolic injury, accelerated aging, cancer, cardiovascular disease, neurodegenerative disease and inflammation [9]. Hence, much attention has focused on the use of antioxidants to protect damage due to free radicals.

Several researchers have repeatedly shown that medicinal plants contain various biologically active secondary metabolites that exert different pharmacological activities: anti-diabetic, antioxidant, anti-inflammatory, analgesic, antitumor, antipyretic, antiplasmodial, antimicrobial, and antiviral, etc. [10]. *Momordica charantia* and *Morinda lucida*, two plants of the Benin pharmacopeia are used traditionally in the treatment of certain diseases.

*M. charantia L* (*Cucurbitaceace*) is a climber growing in tropical regions of Africa. Commonly known as bitter melon, *M. charantia* is cultivated for its use as vegetable as well as medicine [11]. Species of the *Momordica* genus, like as *M. charantia* [12] have been reported to have important hypoglycemic, antidiabetic, antioxidant effects, antiviral, antimalarial and antimicrobial activity [13,14]. Some researchers have attempted to purify the *M. charantia* active fractions and reported that saponins [15], peptides [16] and phenolics [9] extracted from it had the previous cited biological activities.

*M. lucida* (Benth), a member of *Rubiaceae* family, well known by the Fon in southwestern part of Benin as “xwèswè”, is extensively spread in West Africa and is used in African folk medicine to treat several diseases [17]. The leaves are bitter and are used by the natives to medicate malaria, yellow fever, jaundice, hepatitis, eczema, edema, cough, hypertension, diabetes [18,19]. Phytochemical screening of *M. lucida* displayed the existence of important biological active compounds, like tannins, terpenoids, flavonoids and saponins [20]. In Benin, these species have been very little studied, particularly at a medicinal level. The current research desired to analyze polyphenols compounds and investigate antioxidant and enzyme inhibitory activity of *Morinda lucida* and *Momordica charantia* extracts from Benin using microplates to assay extracts. These modified methods incorporate the convenience of spectrometric measurement using 96-well microplates, so that it consumes much less reagents and solvents.

## 2. Materials and Methods

### 2.1. Chemicals

The extraction solvents, p-nitrophenyl-β-D-glucopyranoside (pNPG), α-amylase A3403 Termamyl**^®^** (EC 3.2.1.1) from *Bacillus licheniformis*, β-glucosidase G0395 (EC 3.2.1.21) from almonds, acarbose A8980 and phenolic compounds standards were obtained from Sigma-Aldrich Chemical Company (St. Louis, USA). All the chemicals and reagents utilized were of analytical grade.

### 2.2. Plant Material

The leaves of the plants used were locally grown. *Morinda lucida* leaves samples were collected from Agata (06°30’28’’ N, 002°38’44’’ E), which is located in the department of Oueme, Benin, while those of *Momordica charantia* were collected from Dangbo (06°35’19’’ N, 002°33’15’’ E) located in the same department. A voucher specimens No. AAC8100/HNB and No. AAC8101/HNB respectively for *M. lucida* and *M. charantia* were deposited at the Benin national herbarium, University of Abomey-Calavi, Cotonou, Benin. All samples were collected in the morning at 7 am. They were air-dried (23 ± 2 °C) for two weeks before powdered using grinder Retsch type SM 2000/1430/Upm/Smf, Haan, Germany.

### 2.3. Preparation of Plants Extracts

The samples were prepared by extraction with different polar solvents (water, water-ethanol 30:70 (v/v) methanol, methanol/1% HCl, ethanol, acetone, ethyl acetate, dichloromethane) and non-polar solvents (chloroform and petroleum ether). For the polar solvents, 1 g of powder in 100 mL of solvent was subjected to ultrasonication (35 Hz) at room temperature for 2 h. The same operation was carried out with non-polar solvents under the reflux system. A total of 24 extracts were thus obtained, 12 per plant. In addition, the residues obtained after the ethyl acetate and petroleum ether extractions were extracted again using methanol and methanol/1% HCl respectively. These extracts are coded Methanol-EA and Methanol/HCl-PE. Each mixture was filtered through Whatman N° 1 paper (125 mm ø, Cat No. 1001 125) and concentrated under reduced pressure using a rotary evaporator before oven dried at 40 °C. The aqueous extract was lyophilized to dryness. The extraction yields were determined by the ratio between the mass of powder and extract obtained.

### 2.4. Microplate Determination of Total Polyphenol Content

The total polyphenol content analysis was determined by the Folin–Ciocalteu method adapted after Dicko, 2002 [21] in a 96-well microplate. As a standard reference, a gallic acid solution (0.97–500 µg/mL) was used, and the results presented were expressed as µg equivalents of gallic acid per mg of sample. The absorbance values of the samples were recorded at 760 nm after 30 min of incubation, using a multiwell plate reader (Tecan Pro 200, Tecan Trading AG, Männedorf, Switzerland).

### 2.5. Microplate Determination of Total Flavonoid Content

A modified method after Dah-Nouvlessounon, 2015 [22] was used to quantified the total flavonoid content by a 96-well microplate. The sample absorbance values were read at 415 nm using the Tecan Pro 200 multiwell plate reader. Quercetin was used as a reference standard (0.078–40 µg/mL), and the results are presented as µg equivalents of quercetin per mg of sample.

### 2.6. HPLC-DAD Quantification of Bioactive Polyphenols from M. lucida and M. charantia Extracts

**Figure 1:**
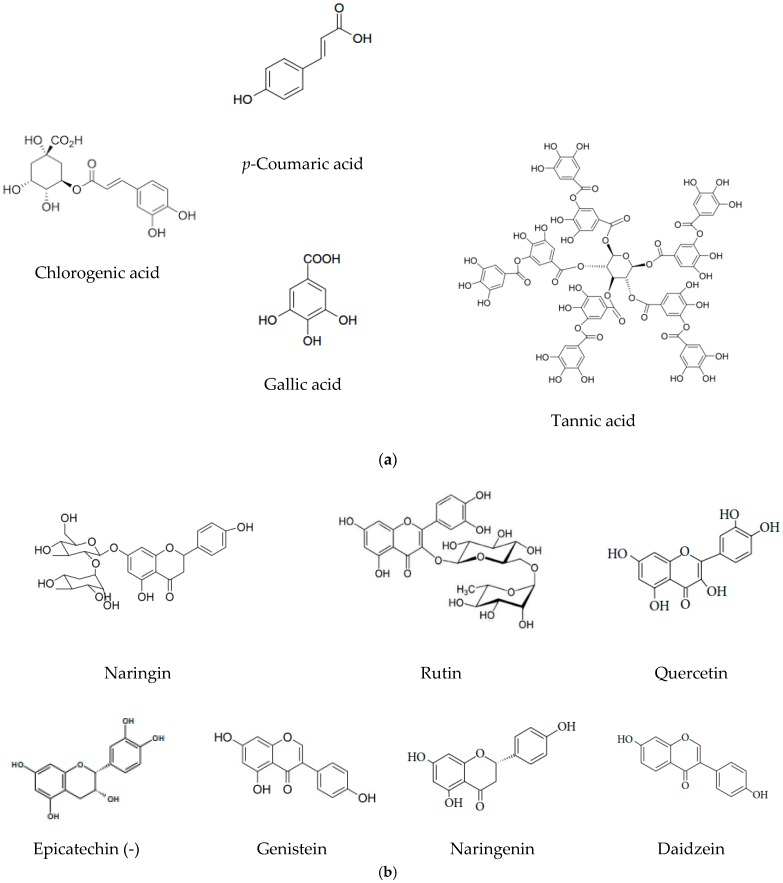
Structure of polyphenolic (**a**) and flavonoid compounds (**b**) identified in *M. charantia* and *M. lucida* leaves extracts.

HPLC-DAD (high-performance liquid chromatography with diode-array detector) investigation was done utilizing a High-Performance Liquid Chromatography Systems L-3000 (RIGOL TECHNOLOGIES, INC Beijing, China). In the chromatographic examination, the Kinetex EVO C18 (150 × 4.6 mm, particle size of 5 μm) column was operated with an injection volume of 10 µL. The solvents used were (A) 0.1% trifluoroacetic acid (TFA) in water and (B) 0.1% trifluoroacetic acid (TFA) in acetonitrile. The gradient elution was for 60 min as 2−100% B at 30 °C and the elution flow was established at 1000 μL/min. Six different analytical wavelengths were used for detection, in accordance with the literature, between λ max 230 and 370 nm. Identification and quantification analyses were performed by comparison with standard spectra at each retention time. Stock solutions of reference compounds as chlorogenic acid, p-coumaric acid, tannic acid, gallic acid, naringin, rutin, quercetin, epicatechin, genistein, naringenin or daidzein (Figure 1) were prepared at the concentration 1000 μg/mL, then for calibration curves, five concentrations were used (10, 50, 100, 200 and 400 μg/mL).

### 2.7. Microplate Determination of Antioxidant Activity 

#### 2.7.1. DPPH radical-scavenging activity

The DPPH (2,2-Diphenyl-1-picrylhydrazyl) method was conducted in accordance with a previous study [23]. This technique hinges on the absorbance minimization at 517 nm, of the DPPH’s free radicals in the presence of H^+^ donor. Based on this assay, were mixed in a 96 well microplate identical volumes of DPPH (50 μM) and plant extracts (200 μg/mL) and allowed at room temperature to stand in darkness for 30 min. At 517 nm the absorbance values were read using a microplate reader (Tecan Infinite M 200 Pro Männedorf, Switzerland). The blank was prepared as the mixture of equal volumes of 100 µL of methanol and DPPH. The DPPH radical’s inhibitory percentage indicates the antioxidant activity of the extracts and standards (ascorbic acid, gallic acid), and was obtained using the formula previously established [24]:

(1) $$\mathrm{Inhibitory}\;\mathrm{Percentage}\;\left(\%\right)=\frac{{\mathrm{Blank}}^{\prime }s\;\mathrm{absorbance}-{\mathrm{Sample}}^{\prime }s\;\mathrm{absorbance}}{\mathrm{Blank}\prime s\;\mathrm{absorbance}\;}\times 100$$

The IC_50_ (concentration providing 50% inhibition) was appraised using mathematical regression. The antioxidant activity index (AAI) was determined in agreement with the formula used in the literature [25].

#### 2.7.2. β-Carotene Bleaching Method

Inhibition of β-Carotene bleaching was determined according to the method characterized in the literature [26]. β-carotene/linoleic acid stock emulsion solution was made by dissolving 4 mg of β-carotene in 20 mL of chloroform, to which 500 μL of linoleic acid and 4 g of Tween 40 were added. Chloroform was entirely evaporated using a vacuum evaporator. Subsequently, 100 mL of miliQ water were added, the resulting emulsion was stirred vigorously. An amount of 50 μL of solubilized extract in methanol (1000 μg/μL) and reference compounds (BHA, rutin and α-tocopherol) at 100 μg/μL, were introduced in a 96-well polypropylene microwell plate. An amount of 250 μL of the above emulsion was added to the extracts and reference compounds samples. A blank consisting of 50 μL of solvent and 250 μL of the emulsion was prepared. The plate was subjected to constant orbital shaking at 660 cycles/min (1 mm amplitude) at 40 °C for the 60 s. A single reading was made at the end of each shaking cycle. The decolorization kinetics of the emulsion in the presence and absence of antioxidant was monitored at 460 nm at time intervals (2, 5, 10, 30, 60, 90, 120, 150 and 180 min) with the same amplitude orbital stirring.

β-carotene bleaching antioxidant activity was calculated as the inhibition percentage of the samples compared to the control, following the formula:

(2) $$\%\mathrm{Inh}=\frac{\mathrm{DRc}-\mathrm{DRs}}{\mathrm{DRc}}$$

DRc: degradation rate of β-carotene in the control sample = {[ln (a/b)]/t}, DRs: degradation rate of β-carotene in the sample with antioxidant = {[ln (a/b)]/t}, a = absorbance at time = 0 min, b = absorbance at defined time (for example at 2,5,10, 30, …., to 180 min), t = time.

### 2.8. In vitro α-Amylase Inhibitory Activity Assay

α-amylase inhibitory activity of plants extracts was accomplished according to the standard technique described in a previous study [27] with minor adjustments. In a 96-well plate, the reaction mixture consisting of 50 μL phosphate buffer (100 mM, pH = 6.8), 10 μL α–amylase: 2 U/mL and 20 μL of varying concentrations of extracts (5, 10, 15, 20 and 25 mg/mL) was preincubated at 37 °C for 20 min. Next, 20 μL of 1% soluble starch (100 mM phosphate buffer pH 6.8) was added as a substrate and incubated additionally at 37 °C for 30 min; 100 μl of the 3,5-Dinitrosalicylic acid (DNS) color reagent was then added and boiled for 20 min. The absorbance of the resulting mixture was assessed at 540 nm using Multiplate Reader (TECAN Infinite M 200 Pro, Männedorf, Switzerland). The known α-amylase inhibitor as acarbose at diverse concentrations was used as a standard. Samples without plant extracts were used as control and each test was carried out in triplicate. The outcome was expressed as percentage inhibition, which was calculated using the following formula:

(3) $$\%\;\alpha -\mathrm{amylase}\;\mathrm{inhibition}\;=\frac{1-\mathrm{As}}{\mathrm{Ac}}\times 100$$

where, As is the absorbance in the presence of extracts and Ac is the absorbance of control.

A dark-blue colour indicates the presence of starch; a yellow colour indicates the absence of starch while a brownish colour indicates partially degraded starch in the reaction mixture. In the presence of inhibitors from the extracts, the starch added to the enzyme assay mixture is not degraded and gives a dark blue colour complex whereas no colour complex is developed in the absence of the inhibitor, indicating that starch is completely hydrolyzed by α-amylase. 

### 2.9. Microplate β-Glucosidase Inhibition Assay

β-Glucosidase inhibitory activity of plant extracts was carried out according to a previous study [28] and adapted in a 96-well plate. Briefly, 20 µL of substrate (p-nitrophenyl-β-D-glucopyranoside, Sigma Chemical Co., 1 mg/mL), 10 µL of varying concentrations of samples (1, 2, 3, 4, 5 and 10 mg/mL) and 20 µL of pH 5 sodium phosphate buffer were mixed in 96-well plate and incubated at 37 °C for 10 min; 10 µL of enzyme solution (β-glucosidase Sigma Chemical Co., 5 mg/mL) were added and the mixture was incubated for another 30 min at 37 °C. 140 µL of pH 10 buffer 50 mM was added to stopped the reaction. Positive control contained, a mixture of solvents instead of the extract; while in the negative control, pH 10 buffer was added at the beginning of the test in order to block enzyme activity. Absorbance was read at 410 nm and the activity was calculated using the following formula:

(4) $$\%\;\mathrm{enzymatic}\;\mathrm{inhibition}\;=100-\left[\frac{\mathrm{Abs}\;\mathrm{test}-\mathrm{Abs}\;\mathrm{negative}\;\mathrm{control}}{\mathrm{Abs}\;\mathrm{positive}\;\mathrm{control}}\times 100\right]$$

### 2.10. Statistical Analysis

The experimental results were presented as mean ± standard deviation (SD) of three parallel measurements. All the graphs were presented using GraphPad Prism 7.00 software. Statistical analyses were performed using one-way analysis of variance followed by Duncan test. *p-*values lower than 0.05 were considered as statistically significant.

## 3. Results

### 3.1. Total Polyphenolic and Flavonoid Contents

The total polyphenolic and flavonoid contents were presented in Table 1. The extract yields of the two medicinal plants vary according to the types of solvent used and also according to the plant species. Indeed, the highest yield of *M. charantia* extracts was obtained using ethanol (23.23 ± 0.45%), while *M. lucida*, water extract gave the highest yield of extraction (19.10 ± 0.10%). On the other hand, petroleum ether is the least extractable solvent for both *M. charantia* (1.03 ± 0.05%) and *M. lucida* (1.43 ± 0.60%). The yield of extraction was not commensurate with phenolic and flavonoid contents. *M. charantia* methanolic (1% HCl) extract showed a high polyphenolic content (6833.88 ± 89.23 µg GAE/mg) which it is two time greater than the total polyphenolic content of *M. lucida* methanolic extract (3048.33 ± 63.63 µg GAE/mg). The highest flavonoid content (692.39 ± 1.89 µg QE/mg) was recorded for *M. charantia* methanolic (1% HCl) extract, and *M. lucida* ethanolic extract (487.41 ± 17.08 µg QE/mg), respectively.

### 3.2. HPLC-DAD Quantification of Bioactive Polyphenols

One of the main objective of this study was the HPLC analysis of polyphenol and flavonoid compounds, abundant micronutrients present in food sources. Different standard compounds namely, tannic acid, gallic acid, chlorogenic acid, epicatechin, p-coumaric acid, daidzein, rutin, naringin, quercetin, naringenin, genistein were used for identification and quantification of bioactive compounds from *M. lucida* and *M. charantia* extracts (Table 2 and Table 3).

**Figure 2:**
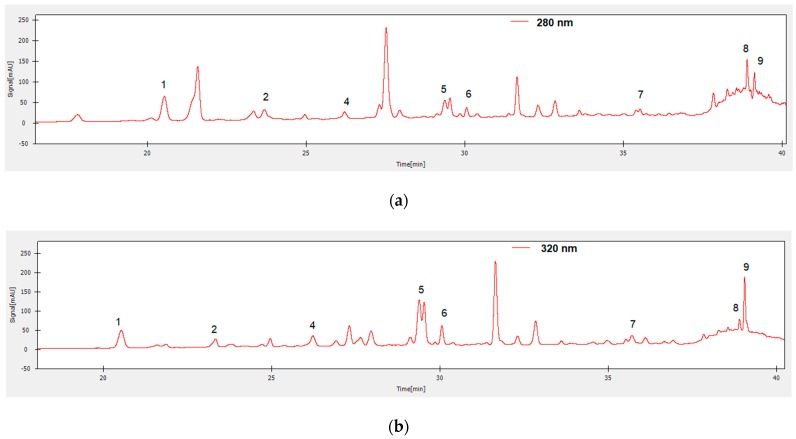
HPLC-DAD chromatograms of *M. lucida* methanol/1% HCl extract with detection at 280 nm (**a**) and 320 nm (**b**). Peaks identified were: 1—chlorogenic acid, 2—epicatechin, 4—daidzein, 5—rutin, 6—naringin, 7—quercetin, 8—naringenin, 9—genistein.

**Figure 3:**
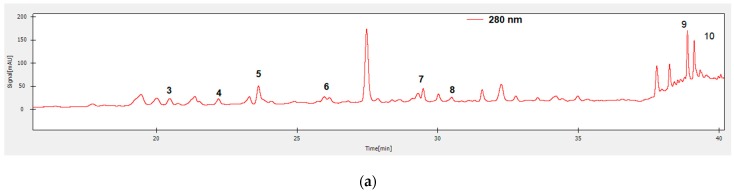

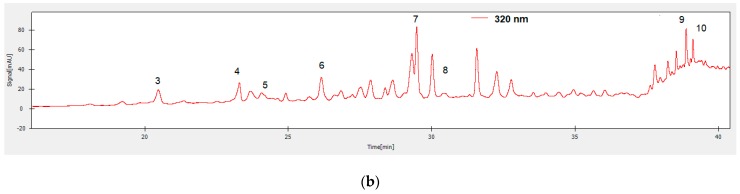
HPLC-DAD chromatograms of *M. charantia* methanol/1% HCl extract with detection at 280 nm (**a**) and 320 nm (**b**). Peaks identified were: 3—chlorogenic acid, 4—epicatechin, 5-p-coumaric acid, 6—daidzein, 7—rutin, 8—naringin, 9—naringenin, 10—genistein.

Figure 2; Figure 3 illustrate the chromatograms of the *M. lucida* and *M. charantia* methanolic (1% HCl) extracts, in which the presence of nine polyphenolic and ten flavonoid and standards, y was observed. The chromatogram of *M. lucida* methanolic (1% HCl) extract (Figure 2) showed the presence, based on the retention time, of chlorogenic acid epicatechin, daidzein, rutin, naringin, quercetin, naringenin and genistein.

The chromatogram of *M. charantia* methanolic (1% HCl) extract showed also peaks which correspond, based on the retention time, to the polyphenolic compound chlorogenic acid and to the flavonoid compounds epicatechin, daidzein, rutin, naringin, quercetin, naringenin and genistein (Figure 3).

The quantification of identified bioactive compounds from *M. lucida* and *M. charantia* extracts was showed in Table 2 and Table 3, respectively. The data from Table 2 shows that 55.55% out of the nine standard compounds were present in *M. lucida* ethanolic and ethyl acetate extracts. *M. lucida* methanolic (1% HCl) extract showed the presence of 88.88% of the standard compounds. Rutin, naringin and genistein have been identified in all *M. lucida* extracts. Naringin is the most abundant compound of *M. lucida* water extracts in a concentration of 730.42 mg/kg. Unlike *M. lucida*, only 10% of the 10 standard compounds were identified in *M. charantia* ethyl acetate extract and 80% were identified in the methanolic (1% HCl) extract (Table 3). Rutin was the compound identified in almost all *M. charantia* extracts, except in the ethyl acetate extract. Moreover, epicatechin (-) has the highest concentration (143.34mg/kg) in *M. charantia* methanolic (1% HCl) extract. 

### 3.3. In Vitro Antioxidant Activities

The evaluation of antioxidant activity of the two plant extracts was assessed in different in vitro models and the extracts displayed various levels of antioxidant activity in all the models studied. The DPPH radical scavenging capacity of the two plant extracts is shown in Table 4 and was expressed as IC_50_ values. The IC_50_ values of *M. charantia* extracts, having inhibited 50% of the DPPH radical, varied from 1.03 ± 0.11 mg/mL (acetone extract) to 25 mg/mL (petroleum ether extract). In addition, the IC_50_ values of *M. lucida extracts*, varied between 0.51 ± 0.01 mg/mL (methanolic extract) and 25 mg/mL (petroleum ether extract). The highest IC_50_ was obtained for non-polar extracts, while the lowest ones were obtained with polar solvents (Table 4). The IC_50_ values of reference compounds, which varied between 0.69 ± 0.01 μg/mL (gallic acid) and 0.38 ± 0.02 μg/mL (ascorbic acid) are lower than those of *M. charantia* and *M. lucida* extracts.

The antioxidant activity index (AAI) value indicates the chance of a compound to be antioxidant, has to have a value lower than 0.5. All the tests showed that the *M. charantia* and *M. lucida* leaves extracts have a low reduction power of DPPH radical. The *M. lucida* methanolic extract showed the greatest potential for reducing the DPPH radical.

**Figure 4:**
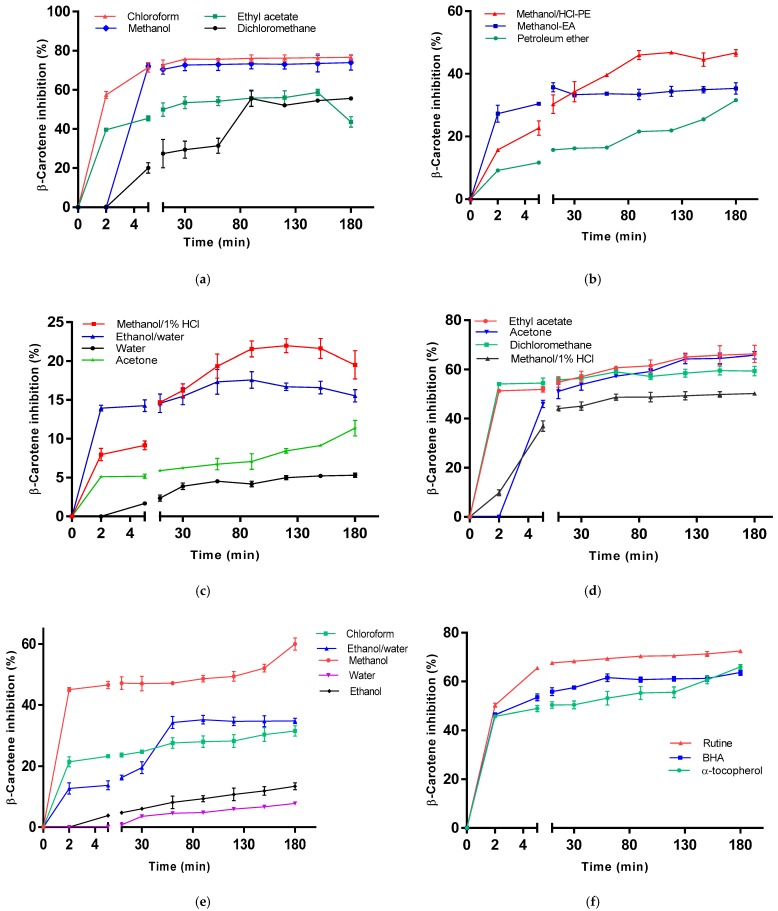
β-carotene bleaching of *M. charantia* extracts (**a**), (**b**), (**c**), *M. lucida* extracts (**d**), (**e**) and standards (**f**).

β-Carotene bleaching assay is likewise used to gauge the antioxidant activity by evaluation of inhibition percent of auto-oxidation of the linoleic acid and β-carotene. The presence of an antioxidant in the *M. charantia* and *M. lucida* extracts will prompt the inactivation of the linoleic acid free radicals. Results presented in Figure 4 showed the highest inhibition percentage (76.60 ± 1.39%), for the *M. charantia* chloroform extract, greater than standard rutin (72.53 ± 0.72%). Similarly, the *M. charantia* methanolic extracts and *M. lucida* ethyl acetate extract showed a higher inhibition percent than standards BHA and α-tocopherol.

### 3.4. Inhibition of α-amylase Activity

**Figure 5:**
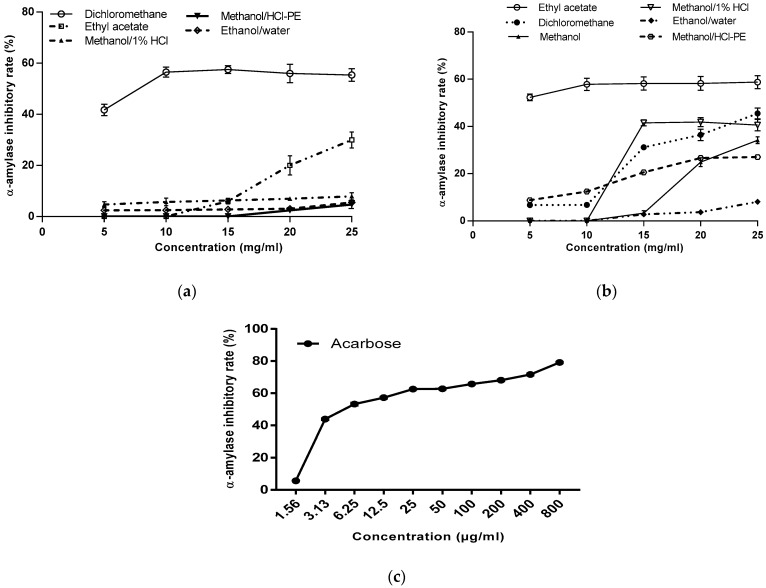
Inhibition of α-amylase activity by *M. charantia* extracts (**a**), *M. lucida* (**b**) extracts and Acarbose (**c**).

The ability to inhibit α-amylase activity has been shown to be dependent on the plant extract compositions, and on the solvent used in the extraction. *M. charantia* showed an inhibitory activity (Figure 5a) when plant was extracted with solvents as dichloromethane, ethyl acetate, methanol/1% HCl, methanol/HCl-PE and ethanol/water. Analysis of variance showed that the interaction between an extract’s activity and concentration is highly variable (*p* < 0.0001). This demonstrates that the inhibition percentage increases with the increases extract’s concentration. The highest inhibition percentage (57.51 ± 0.40%) was obtained for dichloromethane extract at 25 mg/mL their activity presenting IC_50_ values as low as 7.5 ± 0.57 mg/mL. The other *M. charantia* active extracts have an IC_50_ > 25 mg/mL. The comparative action of the extract’s activity according to concentration showed that whatever the concentration (from 5 to 25 mg/mL) the dichloromethane extract showed a highly significant difference (*p* < 0.0001) compared to the other active extracts. Besides no difference (*p* > 0.05) was observed at 15 mg/mL between ethyl acetate, methanol/HCl-PE and ethanol/water extracts. α-amylase inhibition by the *M. charantia* extracts is dose-dependent with low activity.

Concerning *M. lucida* extracts, half of the analyzed extracts showed inhibition of α-amylase activity (Figure 5b). Among these extracts, ethyl acetate extract demonstrated comparatively higher α-amylase inhibitory activities (58.76 ± 2.74%) with IC_50_ value of 5 mg/mL. At 25 mg/mL, the ethanol/water extract had the lowest inhibition percentage (8.10 ± 2.76%). The data analysis revealed that the interaction between the extracts inhibition potency and their concentration is significant (*p* < 0.0001). As shown in Figure 5, degradation of starch by α-amylase was inhibited by the both plants extracts and acarbose used as standard molecule. Whereas, acarbose exhibited stronger inhibitory activity against α-amylase compared to the plants extracts.

### 3.5. In Vitro β-Glucosidase Inhibitory Activity

**Figure 6:**
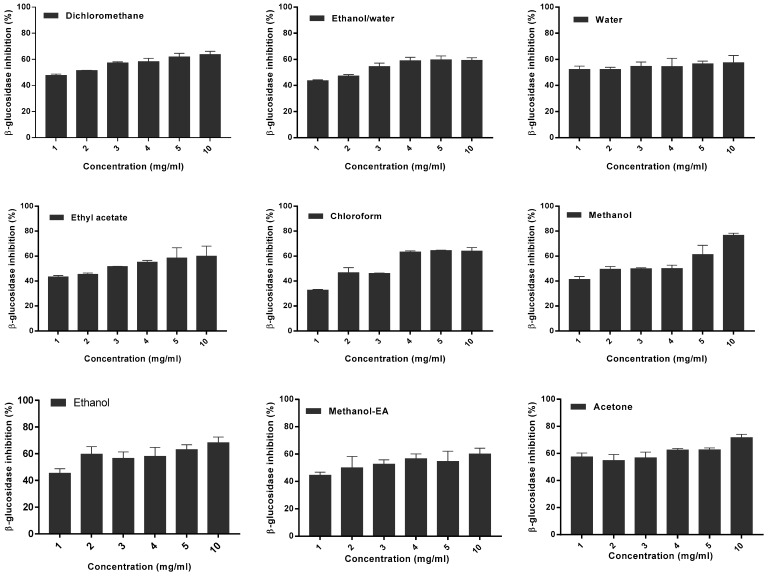

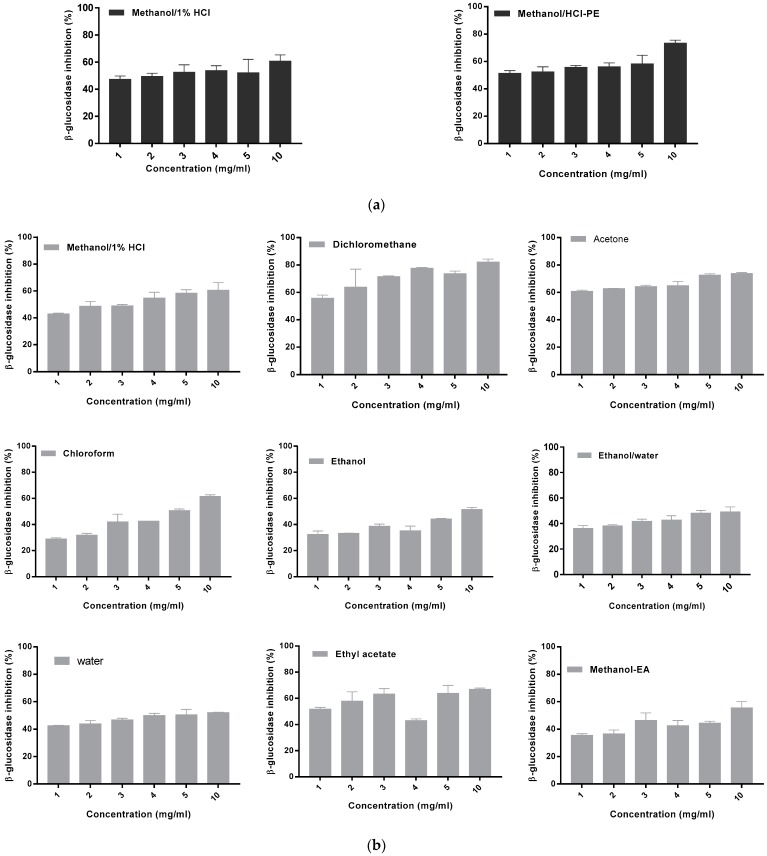
β-glucosidase inhibition activity of the *M. charantia* (**a**) and *M. lucida* (**b**) extracts.

The plant extracts have shown various inhibition percentages of β-glucosidase activity. A significant number of extracts showed an inhibitory activity of 50% or more in dose-dependent manner. Figure 6a shows the results of β-glucosidase activity inhibition by *M. charantia* extracts. The highest inhibitory activity (76.74 ± 1.60%) was presented by the methanolic extract at 10 mg/mL while the lowest (32.73 ± 0.53%) was obtained for the chloroform extract at 1 mg/mL. The data analysis shows that the inhibition percentages of *M. charantia* extracts vary according to the solvent used in extraction (*p* <0.0001). The interaction between the extract types and different concentrations is significant (*p* = 0.0015). 

The inhibition levels of β-glucosidase activity by *M. lucida* extracts are shown in Figure 6b. The highest inhibitory activity (82.11 ± 2.15%) was exhibited by the dichloromethane extract at 10 mg/mL while the lowest (28.95 ± 0.82%) was obtained for the chloroformic extract at 1 mg/mL. The data analysis (Figure 6b) shows that there is a significant variation in the inhibitory activity rate according to the extracts types (*p* <0.0001). For all bioactive extracts, there is a dose response activity which results in the evolution of the percentage of inhibition according to the increase of extracts concentrations. The data showed that the percentage of β-glucosidase activity inhibition increased in both plant extracts when the concentration increased.

## 4. Discussion

All the analyzed samples were prepared by solvent extraction under ultrasound, based on the fact that the mechanical effects of ultrasound induce a disruption of the cell walls, which leads to greater intraparticle penetration of the solvent into the cells, thus facilitating the rapid release of their contents and the acceleration of the kinetics extraction [29]. Ultrasound has the advantage of considerably reducing the extraction time and increasing the extraction yield [30,31]. The yields obtained during the extraction varied from one plant to another and according to the solvents. Since for the same solvent yields vary according to the plant, while the same amount of plant powder was extracted with the same amount of solvent under the same conditions, the explanation of the difference would be related to the chemical composition of the plants that was not be the same. Surely, in the two plants, polyphenol compounds such as, for example, gallic acid, tannic acid, chlorogenic acid, epicatechin, p-coumaric acid, daidzein, rutin, naringin, quercetin, naringenin, genistein were distinguished and evaluated by HPLC-DAD investigation. Kubola and Siriamornpun [9] have also identified gallic acid, tannic acid, *p-* coumaric acid in *M. charantia* leaves extracts using HPLC-DAD analysis. Nagarani et al. [32], Budrat and Shotipruk [33] have identified chlorogenic acid, gallic acid, quercetin and catechin in *M. charantia* leaves extracts in different concentrations. Kazeem et al. [20] have reported that *M. lucida* leaves are a source of phenolic compounds and flavonoids. By this study, we proved that rutin, naringin, naringenin, and genistein, compounds which display good antioxidant, antidiabetic, antiviral and anti-cancer activity, are widely distributed in *M. lucida* extracts, naringin recording the highest concentration (730.42 mg/kg). [34,35]. It has been reported that, in diabetic rats, naringenin reduced diabetic markers through PPARγ and glucose transporter Type 4 (GLUT4) and increased their gene and protein expression levels in pancreas [36]. In the liver, naringenin increased glycogen content, decreased activities of glycogen phosphorylase and glucose-6- phosphatase [37] and ameliorated diabetes induced hepatotoxicity [38]. In the extracts from *M. charantia*, compounds like rutin and daidzein and naringenin are the most common, but epicatechin (-) is the one with the highest concentration (143.34 mg/kg). Ruijters et al. [39] confirmed that o-catechol moiety of (-) epicatechin is essential for the direct detoxifying effects in the reaction with superoxide and hydrogen peroxide [39].

The diversity of these secondary metabolites at the level of each plant gives it a wide range of biological activities. Nowadays, there has been a growing interest in antioxidant, antihyperglycemic, anti-cancer and antiviral agents from natural sources, especially those derived from plants, because they are often considered to be less toxic, with less side effect than synthetic drugs. In the present investigation, some biological activities of *M. lucida* and *M. charantia* extracts were evaluated in vitro.

Indeed, we sought in vitro the inhibitory power of the α-amylase enzyme that would be linked to diabetes disease. One therapeutic approach is the prevention of carbohydrate absorption after food intake, which is facilitated by inhibition of enzymes including α-amylase. In the human species, α-amylase is present in both salivary and pancreatic secretions [4]. This enzyme hydrolyzes complex polysaccharides to produce oligosaccharides and disaccharides which are absorbed through the small intestines into the hepatic portal vein and increase postprandial glucose levels [40,41]. In addition, postprandial hyperglycemia may lead to β-cell dysfunction [42]. What is more, the increased concentration of blood glucose will induce many disorders including retinopathy, nephropathy, neuropathy, and angiopathy. Decreasing postprandial hyperglycemia plays a key role in the treatment of T2DM and pre-diabetic states. Inhibiting carbohydrate-hydrolyzing enzymes in the digestive tract, such as α-amylase contributes to reducing the absorption of glucose, thereby alleviating postprandial hyperglycinemia [43]. In this study, the data demonstrated that some extracts from the two plants (*M. lucida* and *M. charantia*) inhibited α-amylase activity. A dose dependent effect was observed on increasing the concentrations of the extract, suggesting a competitive type of inhibition. Enzyme inhibitors can act according to various mechanisms, combining either with the enzyme (competitive with the substrate or uncompetitive), or with the enzyme-complex substrate (noncompetitive), either with the substrate itself [44]. The enzymatic activity can be specifically affected by many chemical agents and drugs such as acarbose has a form similar to that of oligosaccharides derived from digestion of the starch, it can thus bind to the sites of pancreatic α-amylase, inhibit powerfully, competitively and dose-dependent [45]. Some plants have enzymatic inhibitory activity including polyphenolic compounds and glycoproteins [46]. Many of these polyphenols have an action on α-amylase such as tannins that are able to bind to digestive enzymes and inhibit [47]. *M. charantia* ethyl acetate extract and *M. lucida* dichloromethane extract are shown the best α-amylase inhibitory activity. The α-amylase inhibitory activity in these extracts is most likely to be due to identified compounds in the present study such as: rutin, naringin, naringenin, genistein, chlorogenic acid, epicatechin (-). Hunyadi et al. [48] are showed that chlorogenic acid and rutin play a major role in the in vivo anti-diabetic activity on Type 2 diabetic rats. Naringin and naringenin are reported to have anti-diabetic effects in Type 2 diabetes [37,49]. Genistein has been explored and found to play a protective role against diabetes [50]. For example, the mice treated with 2 mg/kg of genistein had a 55−79% decreased incidence of Type 1 diabetes starting at 14 weeks after exposure [51]. In our study, genistein concentration vary from 1.17 mg/kg to 119.68 mg/kg, which shows that the purified extracts of the two plants could even be used in the treatment of Type 1 diabetes. Besides genistein, (-) epicatechin has a strong antidiabetic activity [52,53]. Considering these reports and the identified compounds in this study, the purified extracts of the both plants could be a good candidate in the treatment of diabetes because inhibition of enzymes involved in the hydrolysis of carbohydrates such as α-amylase has been exploited as a therapeutic approach for controlling postprandial hyperglycemia [54]. The inhibition activity of α-amylase was extended and might be responsible for decreasing the rate of glucose absorption and concentration of postprandial serum glucose [55,56]. This effect would delay the degradation of starch and oligosaccharides, which would in turn cause a decrease in the absorption of glucose and consequently inhibit the increase in postprandial blood glucose [57].

In contrast to the relatively low α-amylase inhibitory potency, extracts from both plants showed high percent of β-glucosidase inhibition ranging from 76.74 ± 1.60% to 82.11 ± 2.15%, respectively, for *M. charantia* and *M. lucida*. It has been reported that in screening plant crude extracts for β-glucosidase inhibitory activity, inhibitory activity of 50% can be considered significant [58]. Apart from the difference in nature between the two enzymes (α-amylase and β-glucosidase), this observation suggests that in our extracts, the molecules involved in the enzymatic inhibition would be the phenolic compounds. In some studies, it is assumed that extracts rich in phenolics have a lower inhibitory effect against amylase activity but a stronger inhibition activity against glucosidase [56]. To testify this hypothesis, a correlation study [6] showed a good relationship between phenolic contents and β-glucosidase activity. Indeed, Table 1 shows that both plants have a good phenolic content range from 113.22 ± 11.46 to 6833.88 ± 89.23 µgEqGA/mg and confirmed by HPLC-DAD analysis. However, we believe that the synergistic action of other molecules contained in our extracts would be the basis of these results because other authors [28] have linked the inhibition of β-glucosidase to the contained alkaloids in their extracts. In addition, fifteen alkaloids are reported to have β-glucosidase inhibition activity, and castanospermine has been identified as the most potent of all [59]. Akpan et al., [60], showed that the alkaloids inhibited the hydrolytic activity of β-glucosidase and Lineweaver–Burk plots showed that the inhibition was a competitive type and Ki values were 0.26 mg/L at inhibitor concentration of 4 mg/L. It is thus not surprising that the two plants are known phenolic and alkaloid producers [61,62]. Therefore, the purified extracts of the two plants (*M. charantia* and *M. lucida*) can potentially be used as an effective therapy for postprandial hyperglycemia with minimal side effects and employed for the control of Gaucher’s disease, related to disturbed lysosomal storage and many diseases. Recent studies have suggested that products which inhibit the activity of β-glucosidase are potentially useful as antiviral, antiadhesive, antibacterial, antimetastatic or immunostimulatory agents [63,64]. Human immunodeficiency virus (HIV), the causative agent of AIDS, contains two heavily glycosylated envelope proteins, gp120 and gp41, and it has been reported that an interaction between glycoprotein gp120 and the cellular protein CD4 is required to initiate the infection cycle. Metabolites capable of inhibiting β-glucosidase activity have shown a possible indirect anti-HIV activity by formally inhibiting glycoprotein processing, which in turn affects the formation of the syncytium and results in an alternative site of action for HIV [54,65,66]. Therefore, the purified extracts of both plants would be good candidates for the treatment of viral diseases as reported by Hernández-Aquino and Muriel [67] with naringenin.

As diabetes and its complications are associated with free radical mediated cellular injury [7]. Reactive oxygen species (ROS) are a class of highly reactive molecules that can cause damage to cells and tissues during various infections and degenerative disorders including cancer [68]. Due to the critical role of scavenging of free radicals including ROS by antioxidants, this property in medicinal plants is considered very relevant. The use of the DPPH radical is a common method to evaluate the antioxidant effect [69]. The results showed with DPPH method the lowest activity of the leaf extracts when compared to the reference molecule such as ascorbic acid (IC_50_ = 0.38 ± 0.02 µg/mL) and gallic acid (IC_50_ = 0,69 ± 0,01 µg/mL). Earlier studies have also shown similar (low) antioxidant activity of these plants leaf and stem bark extracts at concentrations ranging from 5 mg/mL to 50 mg/mL [70,71]. Nevertheless, the IC_50_ obtained in this study show greater activity than those obtained by other authors [9,71]. Besides, *M. lucida* methanolic extract have shown an activity higher than those of gallic acid. HPLC-DAD analysis has shown that *M. lucida* methanolic extract contained the flavonoids who are reported to have the good antioxidant activity like quercetin [72], rutin [73], naringenin [34,67], genistein and daidzein [74].

The presence of different antioxidants can hinder the extent of β-carotene-bleaching by neutralizing the linoleate-free radical and other free radicals formed in the system [75]. Unlike the DPPH method, the chloroform extract prepared in this study had a higher activity than the reference molecules in the β-carotene/linoleate model system. This result can be explained by the polarity of chloroform (apolar solvent). Since the β-carotene bleaching test is similar to a lipid emulsion system in water, Frankel and Meyer [76] have proposed that antioxidants compounds extract by apolar solvent exhibit greater antioxidant properties as they are concentrated within the water. At the lipid-water interface the formation of lipid radicals and the oxidation of β-carotene is thus prevented, while the polar antioxidants remain diluted in the aqueous phase and are thus less effective in the protection of lipids. An extract that delays or inhibits the bleaching of β-carotene can be described as a scavenger of free radicals and as a primary antioxidant [77]. According to several authors, the inhibition test for the oxidation of linoleic acid coupled with that of β-carotene appears to be very useful as a mimetic model of lipid peroxidation in biological membranes [78]. The tested extracts have shown good antioxidant activity with β-carotene bleaching test.

## 5. Conclusions

Given the imperative need to discover new compounds with therapeutic properties, in this study we highlighted compounds with antioxidant properties for various fractions obtained from two plants originating in Benin and we evaluated their antioxidant and antidiabetic activity.

The HPLC data showed that the extracts are rich in phenolic and flavonoid compounds such as chlorogenic acid, epicatechin, daidzein, rutin, naringin, quercetin, naringenin and genistein in different concentrations.

The antioxidant activity assessed by β-carotene bleaching tests was more noteworthy than the decrease of the DPPH radical.

The biologic potential of *M. charantia* and *M. lucida* leaves extracts, focusing on the inhibitory effects on α-amylase and β-glucosidase enzymes was proven to be high, with the second enzyme being more successfully inhibited.

These results partly justify some traditional uses of both plants in the treatment of certain diseases suggesting that *M. charantia* and *M. lucida* leaves may act as immunostimulatory agents, being potential therapeutics for postprandial hyperglycemia and encourage us to continue our work, to exploit the *M. charantia* and *M. lucida* bioactive potential.

## Figures and Tables

**Table 1:** Extract yield and phytochemical composition of plant extracts.

Extracts	*-*	*M. charantia*		*-*	*M. lucida*	
	Extract Yield (%)	Total Polyphenols (µg GAE/mg)	Flavonoids (µg QE/mg)	Extract Yield (%)	Total Polyphenols (µg GAE/mg)	Flavonoids (µg QE/mg)
Water	12.30 ± 0.20	113.22 ± 11.46	65.84 ± 12.07	19.01 ± 0.10	1501.11±76.67	147.32±1.85
Ethanol	23.23 ± 0.45	1853.44 ± 180.99	41.69 ± 2.78	9.63 ± 0.15	2689.11 ± 314.76	487.41 ± 17.08
Ethyl acetate	7.13 ± 0.20	1440.55 ± 21.76	96.33 ± 2.74	4.50 ± 0.55	1173.33 ± 58.16	38.49 ± 3.98
Acetone	9.20 ± 0.30	320.92 ± 8.05	182.20 ± 51.78	6.60 ± 1.15	791.09 ± 24.43	131.33 ± 08.17
Dichloromethane	9.50 ± 0.20	1287.77 ± 26.21	38.45 ± 6.24	7.40 ± 0.85	220.33 ± 88.12	115.86 ± 56.70
Methanol	17.80 ± 0.26	989.55 ± 28.16	123.09 ± 9.63	9.83 ± 1.10	3048.33 ± 63.63	156.71 ± 18.02
Chloroform	5.10 ± 0.36	209.59 ± 31.03	150.78 ± 9.89	2.50 ± 0.70	175.78 ± 22.07	22.35 ± 14.7
Petroleum Ether	1.03 ± 0.05	185.78 ± 32.05	08.96 ± 3.92	1.43 ± 0.60	1353.77 ± 173.05	212.15 ± 57.89
Ethanol/water	22.13 ± 0.15	842.11 ± 52.07	302.28 ± 46.34	17.50 ± 1.17	2184.44 ± 103.21	272.37 ± 66.37
Methanol/1%HCl	18.50 ± 0.10	6833.88 ± 89.23	692.39 ± 1.89	11.23 ± 0.92	906.66 ± 51.47	336.61 ± 15.78
Methanol/HCl-PE	16.30 ± 0.26	705.16 ± 91.21	201.15 ± 1.69	9.60 ± 0.26	2236.11 ± 26.34	191.45 ± 43.82
Methanol-EA	8.80 ± 0.26	700.33 ± 77.66	440.43 ± 25.14	6.73 ± 0.75	2286.00 ± 143.84	441.65 ± d93.37

**Table 2:** HPLC-DAD identification and quantification of polyphenols and flavonoids from *M. lucida* extracts.

Extracts Content (mg/kg)												
Peak	Compound	T_R_ ** (min)	T_R_ * (min)	λ max (nm)	H_2_O	H_2_O-EtOH 30:70	MeOH	MeOH/1% HCl	EtOH	EAC	MeOH-EA	MeOH/ HCl-PE
**1**	**Chlorogenic acid**	20.86	20.88	280, 300	-	-	6.72 ± 0.47	11.72 ± 0.52	-	-	5.31 ± 0.43	8.67 ± 0.52
**2**	**Epicatechin (-)**	23.56	23.74	230, 280	-	107.47 ± 0.72	-	85.44 ± 0.98	71.53 ± 0.83	-	50.59 ± 1.01	74.11 ± 0.14
**3**	**p-Coumaric acid**	24.10	24.22	300, 320	37.68 ± 0.59	52.25 ± 0.63	-	-	-	-	-	-
**4**	**Daidzein**	26.44	26.62	300, 320	15.31 ± 1.02	-	4.49 ± 0.26	19.10 ± 0.01	-	-	7.40 ± 0.70	10.69 ± 0.46
**5**	**Rutin**	29.70	29.68	230, 300	82.02 ± 1.11	74.23 ± 0.03	29.26 ± 0.09	54.53 ± 0.04	23.82 ± 0.07	16.14 ± 0.22	29.19 ± 0.07	28.85 ± 1.16
**6**	**Naringin**	31.51	31.50	320, 370	730.42 ± 38.22	286.31 ± 3.07	45.67 ± 0.02	425.04 ± 1.63	91.37 ± 0.06	3.04 ± 0.03	264.31 ± 1.03	161.17 ± 1.12
**7**	**Quercetin**	37.80	37.97	280, 300	-	13.75 ± 1.04	10.50 ± 2.09	12.76 ± 0.04	-	5.20 ± 1.05	4.94 ± 0.08	-
**8**	**Naringenin**	38.96	38.97	280, 300	50.80 ± 0.10	39.59 ± 1.03	24.39 ± 0.02	27.73 ± 0.05	23.29 ± 1.74	19.82 ± 0.06	-	21.24 ± 1.05
**9**	**Genistein**	39.11	39.19	320, 370	119.68 ± 0.07	57.07 ± 0.22	16.40 ± 0.06	42.80 ± 0.56	15.58 ± 0.13	38.32 ± 0.67	10.36 ± 0.03	16.93 ± 1.53

** Retention time (TR) mean error for standard references was ± 0.0001–0.2 min. * Retention time (T_R_) mean error for compounds was ± 0.0001–0.2 min. - not detected, MeOH: methanol, EtOH: ethanol, EAC: ethyl acetate, PE: petroleum ether.

**Table 3:** HPLC-DAD identification and quantification of polyphenols and flavonoids from *M. charantia* extracts.

Extracts Content (mg/kg)												
Peak	Compound	T_R_ ** (min)	T_R_ * (min)	λ max (nm)	H_2_O	H_2_O-EtOH 30:70	MeOH	MeOH/1% HCl	EtOH	EAC	MeOH -EA	MeOH/ HCl-PE
**1**	**Tannic acid**	2.30	2.32	280	18.74 ± 0.04	-	-	-	-	-	-	-
**2**	**Gallic acid**	3.51	3.31	250	30.21 ± 0.23	-	-	-	-	-	-	-
**3**	**Chlorogenic acid**	20.86	20.93	280, 300	7.24 ± 1.02	-	-	11.35 ± 0.20	-	-	-	0.97 ± 0.01
**4**	**Epicatechin (-)**	23.56	23.68	230, 280	-	-	-	143.34 ± 0.90	-	55.67 ± 0.44	-	12.49 ± 0.09
**5**	**p-Coumaric acid**	24.10	24.10	300, 320	-	65.88 ± 0.05	-	55.12 ± 0.57	-	-	-	4.86 ± 0.02
**6**	**Daidzein**	26.44	26.48	280, 320	-	9.37 ± 0.04	10.16 ± 0.15	20.82 ± 0.01	10.06 ± 0.52	-	3.56 ± 0.02	1.83 ± 0.05
**7**	**Rutin**	29.70	29.71	320, 370	21.88 ± 0.38	38.61 ± 0.06	41.56 ± 0.47	45.75 ± 0.46	40.87 ± 1.05	-	20.54 ± 0.10	3.83 ± 0.04
**8**	**Naringin**	31.51	31.72	320	-	-	-	1.07 ± 0.02	-	-	-	-
**9**	**Naringenin**	38.96	38.96	280, 300	29.85 ± 0.53	-	38.89 ± 0.36	45.44 ± 0.09	-	-	19.17 ± 0.38	3.96 ± 0.02
**10**	**Genistein**	39.11	39.20	300, 320	36.02 ± 0.15	-	-	54.77 ± 0.04	-	-	-	1.17 ± 0.02

** Retention time (TR) mean error for standard references was ± 0,0001–0,2 min. * Retention time (T_R_) error of mean for compounds was ± 0,0001–0,2 min. - not detected, MeOH: methanol, EtOH: ethanol, EAC: ethyl acetate, PE: petroleum ether.

**Table 4:** DPPH radical-scavenging activity.

Extracts Type	*M. charantia*		*M. lucida*	
	IC_50_ (mg/mL)	AAI	IC_50_ (mg/mL)	AAI
Water	>10	nd	3.35 ± 1.20	0.01 ± 0.11
Ethanol	1.24 ± 0.07	0.04 ± 0.01	1.53 ± 0.37	0.03 ± 0.00
Ethyl acetate	1.25 ± 0.21	0.04 ± 0.02	7.02 ± 09	0.01 ± 0.00
Acetone	1.03 ± 0.11	0.05 ± 0.02	0.91 ± 0.02	0.05 ± 0.00
Dichloromethane	>10	nd	>10	nd
Methanol	1.30 ± 0.12	0.03 ± 0.01	0.51 ± 0.01	0.10 ± 0.00
Chloroform	6.95 ± 0.21	0.01 ± 0.00	>10	nd
Petroleum Ether	>25	nd	>25	nd
Ethanol/water	2.36 ± 0.08	0.02 ± 0.00	1.00 ± 0.00	0.05 ± 0.00
Methanol/1%HCl	1.14 ± 0.02	0.04 ± 0.00	6.05 ± 0.13	0.01 ± 0.07
Methanol/HCl-PE	3.60 ± 0.26	0.01 ± 0.00	-	-
Methanol-EA	1.33 ± 0.11	0.03 ± 0.01	-	-
Reference compound	IC_50_ (µg/mL)	AAI	-	-
Ascorbic acid	0.38 ± 0.02	130.57 ± 5.14	-	-
Gallic acid	0.69 ± 0.01	71.78 ± 1.17	-	-

AAI: antioxidant activity index, nd: not determined.
